# Investigation of the Effects of Blocking Potassium Channels With 4‐Aminopyridine on Paclitaxel Activity in Breast Cancer Cell Lines

**DOI:** 10.1002/cnr2.70072

**Published:** 2024-12-08

**Authors:** Esra M. Cüce‐Aydoğmuş, G. Ayşe İnhan‐Garip

**Affiliations:** ^1^ School of Medicine, Biophysics Department T.C. Maltepe University Maltepe İstanbul Turkey; ^2^ School of Medicine, Biophysics Department T.C. Marmara University Maltepe İstanbul Turkey

**Keywords:** apoptosis, breast cancer, cell cycle, drug mechanism, drug repurposing, transmembrane potential

## Abstract

**Background:**

Paclitaxel (PTX) has been used as a chemotherapeutic agent for several malignancies, including breast cancer, and efforts to increase the efficiency of PTX are continuous. Previous studies have shown that the voltage‐gated K^+^ channels are over‐expressed in breast cancer cell lines; therefore, blocking this type of K^+^ channel reduces cell proliferation and viability.

**Aims:**

In this study, FDA‐approved 4‐aminopyridine (4‐AP), a voltage‐gated potassium channel blocker, was used in combination with PTX to improve the anticancer activity of PTX in MCF‐7 and MDA‐MB‐231 cell lines.

**Methods and Results:**

Viability was determined with trypan blue, a clonogenic assay was performed, and the cell cycle was determined with a flow cytometer and immunochemistry. To gain an insight into the mechanism, intracellular K^+^ concentration, intracellular Ca^2+^ (calcium) concentration, and transmembrane potential measurements were made with corresponding fluorescent dyes. The apoptotic cell number was determined using Annexin /PI method by flow cytometer. Viability decreased with combination therapy and the clonogenic assay proved decreased colony formation. The apoptotic cell number was increased after treatment with the combination in both cell lines. Cell cycle measurements showed G_1_ arrest for both MCF‐7 and MDA‐MB‐231 cell lines upon 4‐AP treatment. PTX caused G_1_ arrest in MCF‐7 cells and S phase arrest in MDA‐MB‐231 cells. Combination treatment caused S phase arrest in MCF‐7 cells and S phase and G_2_/M phase arrest in MDA‐MB‐231 cells. Intracellular K^+^ concentration was increased after all treatments in both cell lines. Ca^2+^ concentration was increased significantly after combination treatment. Depolarization in the transmembrane potential was observed after all treatments in both cell lines.

**Conclusion:**

Biophysical parameters like the transmembrane potential and ion fluxes have been defined in cancer progression which can provide new aspects for cancer treatments. This study shows that the combination of 4‐AP with PTX is a promising alternative the mechanism of which needs further investigation considering the results obtained for Ca^2+^, K^+^, and membrane potential.

## 1 Introduction

According to the data of the World Cancer Research Fund International, breast and lung cancers are the most common types of cancer. The prevalence of breast cancer (BC) among women worldwide is 11.7% of total cancer cases, and 24.5% of the cancers in females and with this rate, breast cancer ranks first [1]. The molecular classification involves three major subtypes depending on estrogen or progesterone receptor expression and human epidermal growth factor 2 (ERBB2; formerly HER2): hormone receptor‐positive/ERBB2 negative and exhibits different histopathological, biological features and thus clinical outcomes [2].

Paclitaxel (Taxol; PTX) is a pseudoalkaloid that was accepted as a chemotherapeutic drug by the FDA for ovarian cancer in 1973 and for BC in 1974 [3]. Since then, it has been effectively used for ovarian cancer, nonsmall lung cancer, head and neck tumors, Kaposi's sarcoma, and urologic malignancies albeit its side effects like peripheral neurotoxicity, neutropenia, leukopenia and hypersensitivity due to cremophore, the vehicle used to deliver PTX [4]. Paclitaxel belongs to the family of microtubule‐targeting drugs. It is generally accepted that PTX acts on microtubule dynamics, promotes polymerization, and thus blocks the cell cycle at G_2_/M causing cell death by activating the Spindle Assembly Checkpoint (SAC) [5, 6].

On the other hand, it has been shown that the effect of PTX on the cell cycle is concentration‐dependent and a G_1_ block is observed at lower concentrations [7, 8]. Besides it has been shown that PTX affects Bcl‐2 phosphorylation, induces Ca^2+^ release from mitochondria thus initiating apoptotic cascade, and has immunomodulatory effects via regulation of chemokines, cytokines, or immune cells. (For an extensive review see Abu Saaman et al., 2019; Kampan et al., 2015).

Although quite effective and widely used BC can develop resistance to paclitaxel. This is a major problem causing the poor response, tumor recurrence, and metastases and represents the leading cause of mortality in BC. The treatment regimen is usually planned as combination therapy for higher effectiveness, the most frequently used being Doxorubicin, cisplatin, and trastuzumab [9].

We confronted this problem from a different angle. First: Voltage‐gated potassium ion channels which are the most diverse group of ion channels have been implicated in carcinogenesis due to the observation that their expression is dysregulated in cancer [10, 11]. Since a substantial amount of research has revealed decreased proliferation and cell death by blocking K^+^ channels [12, 13, 14, 15], they are considered to be potential targets for cancer therapy [16, 17]. Secondly, although proliferating cells like stem cells, embryonic cells, and cancer cells are more depolarized compared to differentiated cells [18], during the cell cycle, they undergo transmembrane potential (Vm) cycles; depolarization at the G_0_/G_1_ phase, hyperpolarization at the G_1_/S phase, and a consequent depolarization at the G_2_/M phase [19, 20]. Failure of these potential alterations which the voltage‐gated potassium channels (VGKC) are mainly responsible for, causes the cell cycle to halt. Considering the promising importance of targeting the K^+^ channel we hypothesized that 4‐aminopyridine (4‐AP), a voltage‐gated K^+^ channel blocker would increase the efficacy of PTX.

4‐AP is an FDA‐approved drug that is currently used for neurological disorders like multiple sclerosis and chronic spinal cord injury [21]. On the other hand, 4‐AP has also been seen to have proapoptotic properties and suppress proliferation at commonly used concentrations [22, 23]. 4‐AP by causing depolarization activates calcium entry which in turn would lead to apoptosis [23]. In human lymphocytes, membrane depolarization induced by 4‐AP results in an elevation of intracellular pH and an increase in intracellular calcium [24]. 4‐AP induced a significant increase in intracellular Ca^2+^ concentration through the P2X7 receptor [25, 26]. Wang et al. have stated that 4‐AP arrests the cell cycle at the G_1_ phase probably by depolarizing Vm thus blocking hyperpolarization needed to pass to the S phase, ultimately leading to cell death by the influx of calcium [27].

In this study, we aimed to increase the effectiveness of paclitaxel, in combination with 4‐AP. MCF‐7 and MDA‐MB‐231 cells were treated with PTX and 4‐AP combination and then cell viability, colony formation, apoptosis, cell cycle determination, intracellular Ca^2+^ and K^+^ concentration, and finally, the transmembrane potential was determined.

## 2 Materials and Methods

### 2.1 Cell Lines and the Growth Condition

The BC cell lines MCF‐7 (ATCC HTB 22) and MDA‐MB‐231(ATCC HTB 26) were used in this study. The MCF‐7 cell line is ER^+^ and PR^+^ and MDA‐MB‐231 cell line is triple‐negative, ER^−^, PR^−^, and HER2^−^ and considered to be metastatic. Cells were grown in DMEM medium containing L‐glutamine (Capricorn Scientific GmbH, Germany) supplemented with 10% fetal bovine serum (FBS; Capricorn Scientific GmbH, Germany), 1% penicillin/streptomycin (Capricorn Scientific GmbH, Germany) in a humidified atmosphere containing 5% CO_2_ at 37^o^C.

### 2.2 Cell Viability and Cytotoxicity of PTX and 4‐AP: Determination of IC_50_ Values

PTX (Sigma Aldrich St. Louis MO, USA) and 4‐ AP (Sigma Aldrich St. Louis MO, USA) stock solution was prepared by dissolving in DMSO (Sigma Aldrich St. Louis MO, USA) and stored at −20^o^C. A growth medium was used for preparing the desired concentration from the stock solution. The final concentration of DMSO was 0.01% and the control groups were with and without DMSO. From a cell survival point of view, no difference was observed between the control without DMSO and the control with DMSO.

At first cytotoxicity determination was planned to use the MTT (Thermo Fisher, Massachusetts, USA) method but PTX and 4‐ AP by generating reactive oxygen species interfered with MTT (Ulukaya et al. 2008) resulting in false positive results. Therefore, determinations of viability and IC_50_ values were performed by the trypan blue exclusion method on the hemocytometer. MCF‐7 and MDA‐MB‐231 cells were seeded on 6‐well plates, cell numbers were 1.8 × 10^5^ cells/well in 3 mL growth medium, and incubated overnight in a humidified atmosphere containing 5% CO_2_ at 37^o^C. After incubation, cells were treated separately with different concentrations of PTX and 4‐AP and incubated for 24 h and then counted with a hemocytometer. Results were compared with control groups.

### 2.3 Colony Formation Assays

Cells were plated in 6‐well plates at a density of 1.8 × 10^5^ cells/well, 3 mL/well. Following overnight incubation, cells were treated with IC_50_ dose of 4‐AP, PTX, and their combination and incubated for 24 h. After incubation, cells were trypsinized with trypsin EDTA (Pan Biotech GmbH, Aidenbach, Germany) and counted with a hemocytometer. Cells were then re‐seeded in 6‐well plates at a density of 500 cells /well for MCF‐7, and 1000 cells/well for MDA‐MB‐231 in 2 mL/ well growth medium followed by an additional 15 days incubation to allow colonies to form. During colony growth, the culture media were replaced every 3 days. 15 days after seeding colonies were fixed with methanol and stained with 0.1% crystal violet, washed to remove the excessive dye, and counted under invert microscopy. A colony was counted only if it contained more than 50 cells.

### 2.4 Measurements of the Apoptotic Cell Numbers

IC_50_ (7.5 nM for PTX and 4 mM for 4‐ AP) values were used throughout the experiments. Apoptosis measurements were performed using an Apopnexin FITC apoptosis detection kit (Millipore, Billerica, MA, USA) by flow cytometer (BD Facs Calibur, Becton, and Dickinson, San Jose, CA, USA). After the combination treatments, apoptosis measurements were performed according to the manufacturer's protocol. The results of apoptosis measurements were analyzed by the Cell Quest program.

### 2.5 Determination of the Intracellular K^+^ Concentration

Intracellular potassium concentrations were determined with PBFI‐AM (Molecular Probes, Invitrogen, Carlsbad, CA, USA) dye, which is sensitive to intracellular potassium, by a microplate reader (Synergy H1, BioTek Instruments, VT, USA). PBFI‐ AM was dissolved in DMSO. The stock solution was diluted with HBSS (Gibco, GIBCO–Invitrogen, Carlsbad, CA, USA). Cells were seeded in 96 wells of black plate with cell numbers 50 × 10^3^ wells in 100 μL growth medium and incubated overnight. Then drugs were added to wells and incubated for 24 h. Following incubation, the growth medium containing the drug was removed and cells were washed two times with HBSS. PBFI‐ AM was added in wells with a final concentration of 5 μM in HBSS and incubated for 1 h. The cells were then washed two times with HBSS to remove an excessive amount of dye. Fluorescent measurements were taken in the HBSS medium. The excitation wavelength was 340 nm; the emission wavelength was 505 nm.

### 2.6 Determination of the Intracellular Ca^+2^ Concentration

Changes in intracellular Ca^+2^ concentration were determined using Screen quest FURA‐2 no‐wash calcium assay kit (AAT Bioquest Inc., Pleasanton, CA, USA) by a microplate reader (Synergy H1, BioTek Instruments, VT, USA). Fura‐2 was dissolved in DMSO. Measurements of intracellular Ca^+2^ were taken according to the manufacturer's protocol. Cells were seeded in 96 wells black plate; cell numbers were 10^4^ cells/ well in 100 μL. Excitation wavelengths were 340 and 380 nm, emission wavelength was 510 nm. The measurements were conducted for 24 h immediately after drugs had been added.

### 2.7 The Transmembrane Potential Measurements

The transmembrane potential was determined with Bis‐(1,3‐ dibutyl barbituric acid) trimethine oxonol (DiBAC_4_(3); AAT Bioquest Inc., Pleasanton, CA, USA). Fluorescence dye by a microplate reader (Synergy H1, BioTek Instruments, VT, USA). DiBAC_4_(3) is in the class of hydrophobic slow‐response dyes that can pass through the cell membrane. When the cells are incubated with the dye, DiBAC_4_(3) due to its hydrophobic nature migrates from the extracellular aqueous medium to the lipid membrane. Unlike cationic and carbinacyanin dyes, DiBAC_4_(3) is not absorbed by mitochondria and its main selectivity is the membrane potential. DiBAC_4_(3) was dissolved in spectroscopic DMSO, concentration value was 20 mM. Cells were seeded in 96 wells black plate, and the cell number was 50 × 10^3^ cells/ well in 100 μL growth medium and incubated overnight. Following incubation, the medium was removed, and 100 μL HBSS was added per each well. Drugs were added in wells and incubated for 2 h. Following incubation, DiBAC_4_(3) solution was added in wells, the final concentration was 2 μM in 100 μL HBSS incubated for 30 min and then, to remove an excessive amount of dye, cells were washed with HBSS and drug treatment was repeated. Measurements were taken in an HBSS medium, the excitation wavelength was 540 nm, and the emission wavelength was 590 nm. To determine changes in fluorescent intensity, measurements were taken at a time interval of 30 min for 24 h.

### 2.8 Determinations of the Cell Cycle Arrests

Two cell cycle analyses were made. Cell cycle assay was performed using a cell cycle assay kit (Abcam, Cambridge, UK) with quantitative immunocytochemistry to measure levels of Cdk2 protein phosphorylated Tyr15 (elevated in the G_1_/S arrest) and histone H3 protein phosphorylated Ser10 (elevated in G_2_/M arrest) levels. Cells were seeded in the 96‐well black wall plate. The number of cells was 25.000 cells/well in each well. Cell number was chosen according to the doubling time of the cell and the wall surface area. Cell cycle measurements were taken according to the manufacturer's kit protocol using a microplate reader (Synergy H1, BioTek Instruments, VT, USA).

For detailed analysis, a flow cytometer was used as the second method. Cell cycle analyses were performed with RNAse A and PI in a flow cytometer to detect cell cycle arrests after drug treatments. The cells were seeded in 6‐well plates the day before the drug treatments at 1.8 × 10^5^ cells in 3 mL of medium per well, incubated overnight in a CO_2_ humidifier at 37^o^C for adhesion. Drugs were added to the wells and incubated in a CO_2_ humidifier at 37^o^C for 24 h. After incubation, cells were fixed using 70% ethanol and incubated at −20°C for 12 h. After fixation, it was washed two times with PBS. After washing, it was incubated with PBS containing 100 μg/mL RNAse for 30 min at 37°C. It was stained with 50 μg/mL PI to mark intracellular DNA. Measurements were taken on a flow cytometer (BD Facs Calibur, Becton and Dickinson, San Jose, CA, USA), and analyses were performed using Cell Quest software.

### 2.9 Statistical Analyses

Data from this study were generated from at least three independent experiments. Results were represented as mean ± SD (standard deviation) in the figures. Statistical analysis was performed with a two‐sided independent Student's *t*‐test to compare two means, and a one‐way analysis of variance (ANOVA) to compare more than two means of one variable. As posthoc analysis (multiple comparisons), Dunnett's test was performed for IC_50_ determination and analysis of the other experiments, and Tukey's test was performed as posthoc analysis. For all analyses, *n* ≥ 3, differences with *p* < 0.05 were considered statistically significant and indicated with *, *p* < 0.01 was indicated **, *p* < 0.001 was indicated ***, and *p* < 0.0001 was indicated with ****. Detailed statistical evaluations are represented in the Data S2.

## 3 Results

### 3.1 The Combination Treatment of 4‐AP and PTX Caused a Reduction in Cell Viability and Colony Formation Ability, and an Increase in Apoptotic Cell Number

**FIGURE 1:**
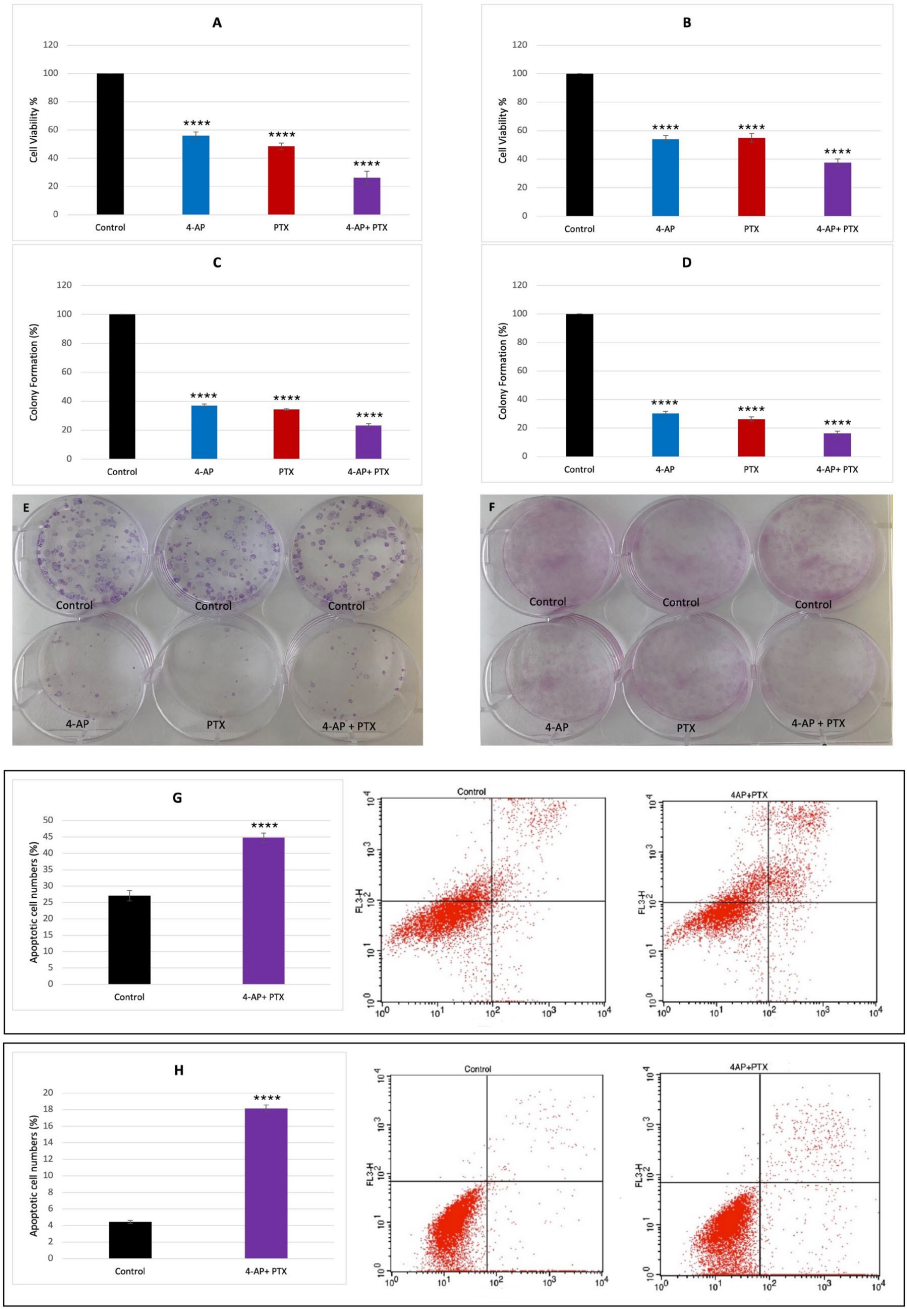
Cell viability, colony formation and apoptotic cell number. Cell survival was determined by the trypan blue method on the hemocytometer. For 4‐ AP, the 4 mM concentration value was determined as IC_50_ and this concentration value was used in combination treatments for both cell lines. For PTX, 7.5 nM was found as IC_50_ for the MCF 7 cell line and 8 nM was found as IC_50_ for the MDA‐MB 231 cell line. A 7.5 nM concentration value was selected for the combination treatments. The survival comparison between separate drug treatments and combination treatment in the MCF‐7 cell line (A), and the MDA‐MB 231 cell line (B). Colony formation was performed after treatments, colony number decreased after combination treatment in MCF7 (C, E) and in MDA‐MB 231cell lines (D, F). Apoptosis determination was performed using Annexin/PI method by flow cytometer. Apoptotic cell numbers increased after combination treatment in MCF‐7 (G) and MDA‐MB‐231 cell lines (H). For all experiments, *n* ≥ 3, *p* < 0.0001 was indicated with **** (with respect to control).

To determine the cytotoxicity of 4‐AP, a literature review was first performed, and the concentration ranges were selected as 1 mM–5 mM (Data S1). The results of the viability determinations were made after 24 h of incubation with the determined concentration values. The IC_50_ value of 4‐AP was found to be 4 mM for both cell lines. A concentration range of 1–10 nM was selected to determine IC_50_ values of PTX. Cells were incubated for 24 h. The IC_50_ value of PTX was determined to be 7.5 nM for MCF‐7 and 8 nM for the MDA‐MB‐231 cell line. A dose value of 7.5 nM was chosen for the combination experiments for both cell lines. To reveal the effect of the 4‐AP on PTX activity, viability determinations were made by treating the cells with 4 mM 4‐AP and 7.5 nM PTX for 24 h. It was seen that the combined use of 4‐AP and PTX caused a 73.7% ± 4.5% (*p* < 0.0001) decrease in cell viability in MCF7 cells—which is in accordance with our previous experiments [28] and a 62.4% ± 2.51% (*p* < 0.0001) decrease in MDA‐MB‐231 cells (Figure 1A,B).

To evaluate the effects of the drugs after they were removed from the cell media, a colony‐forming assay was performed to determine whether the decrease in cell viability was reversible. Treatments of the 4‐AP, PTX, and their combination effects were evaluated. It was observed that although 4‐AP and PTX treatments caused a decreased colony formation, combination treatment of these drugs resulted in a more effective decrease in colony formation. 4‐AP, PTX, and their combination caused a reduction in the colony number of 63.4% ± 2.24% (*p* < 0.0001), 65.5% ± 1.82% (*p* < 0.0001), and 72.7% ± 0.96% (*p* < 0.0001) respectively, relative to control in the MCF‐7 cell line (Figure 1C), in the MDA‐MB‐231 cell line, the reduction of the colony number was 70.3% ± 1.58% (*p* < 0.0001) for 4‐AP, 76.0% ± 5.37% (*p* < 0.0001) for PTX and 85.9% ± 4.62% (*p* < 0.0001) for the combination treatment relative to control (Figure 1D). The data from statistical analysis showed that in both cell lines, the whole comparisons were significant, and the effect of the combination treatment on colony formation was significantly different from separate treatments. This means that the effects of drugs on cell viability and ability of colony formation were not reversible, this combination treatment caused a decrease in the rate of cell proliferation.

To reveal which cell death mechanism underlies the decrease in the cell viability and the colony number after combination treatment, apoptosis measurements were taken with Annexin V and PI method with flow cytometry. After the combination treatment, the increase in the number of apoptotic cells in MCF‐7 and MDA‐MB‐231 cells compared to the control was determined as 44.8% ± 1.3% (*p* < 0.0001) for MCF‐7% and 18.15% ± 0.4% (*p* < 0.0001) for MDA‐MB‐231 (Figure 1E,F).

### 3.2 Treatments of 4‐AP, PTX, and the Combination Increased Intracellular K^+^ Concentration

**FIGURE 2:**
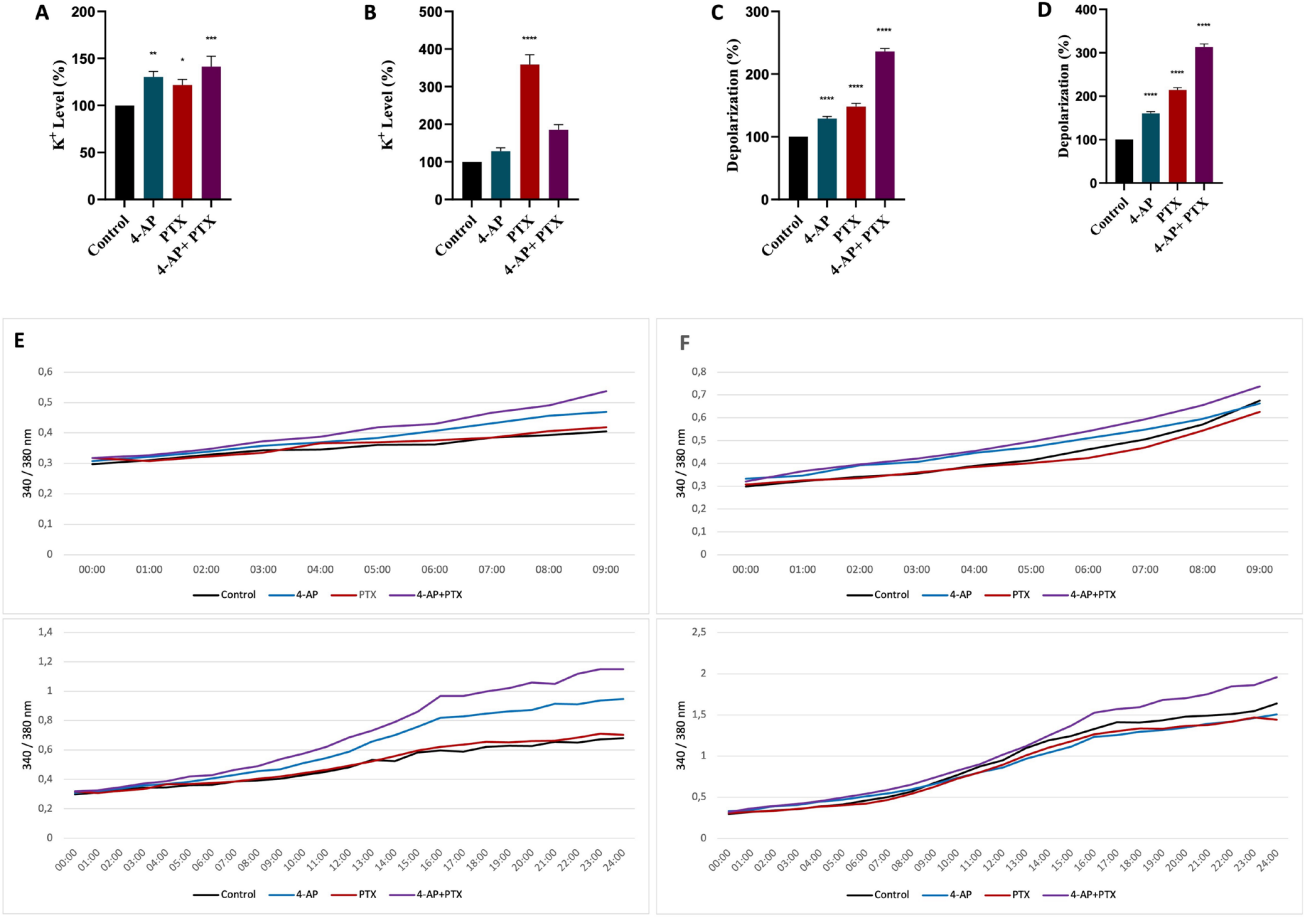
The changes of intracellular K^+^ levels, intracellular Ca^2+^ concentration, and depolarization measurements of the transmembrane potential. After the treatments with 4‐AP, PTX, and the combination for 24 h, intracellular K^+^ was measured with PBFI/AM fluorescent dye by the fluorescence spectrophotometer. For all treatments, intracellular K^+^ levels increased with different ratios: MCF‐7 (A) and MDA‐MB‐231 (B) where the figures are represented with respect to control. After all treatments, determination of the intracellular Ca^2+^ was performed by Fura2 fluorescent dye. In MCF‐7 (E) and MDA‐MB‐231 (F) cell lines. To determine the depolarization of the transmembrane potential, a DiBAC_4_(3) fluorescent indicator was used. After all treatments, depolarization of the transmembrane potential was observed in the MCF‐7 cell line (C) and MDA‐MB‐231 (D) cell line. For all experiments, *n* ≥ 3, p < 0.05 was indicated *, *p* < 0.01 was indicated **, *p* < 0.001 was indicated ***, and *p* < 0.0001 was indicated with **** (with respect to control).

The overexpression of voltage‐gated K channels in BC cell lines was demonstrated. To assess the channel blocker activity of 4‐AP, both separately and in combination with PTX, intracellular K^+^ concentrations were measured using the PBFI‐AM ELISA kit. In the MCF‐7 cell line, 4‐AP, PTX, and the combination caused an increase of 30.33% ± 5.85% (*p* < 0.01), 21.67% ± 6.02% (*p* < 0.05), and 41.33% ± 11.15% (*p* < 0.001) respectively (Figure 2A). In the MDA‐MB‐231 cell line, the application of 4‐AP caused a 28.67% ± 8.5% increase which is similar to the increase in the MCF‐7 cell line. However, in the MDA‐MB‐231 cell line, PTX and 4‐AP + PTX seemed to cause a drastic increase of 258.7% ± 26.3% (*p* < 0.0001), and 81% ± 14.1% (*p* < 0.001) respectively (Figure 2B). In the MCF‐7 cell line, changes in intracellular K^+^ concentration were statistically significant. The effects of each drug on intracellular K^+^ concentration were distinct, with all contributing to an increase in K^+^ levels.

In the MDA‐MB‐231 cell line, an increase in intracellular K^+^ concentration was anticipated after 4‐AP and combination treatments. The combination treatment and PTX affected intracellular K+ concentration differently; specifically, PTX alone led to an increase in K^+^ concentration in the MDA‐MB‐231 cell line which was unexpected, indicating that PTX caused K^+^ accumulation specifically in this cell line (see Data S2).

### 3.3 After all Treatments, Intracellular Ca^+2^ Concentration Changed With a Different Pattern

The “Screen Quest Fura‐2 AM No Wash Ca assay kit” used in the experiments allows homogeneous measurement of intracellular Ca^2+^ change caused by the activation of Ca^2+^ channels or G protein‐coupled receptors. Ca^+2^ changes were monitored depending on time and by measuring for 24 h after the drugs were added.

In the MCF‐7 cell line, after the 4‐AP and the combination treatments, an increase was observed of 26% ± 1.5% and 45% ± 1.5% respectively from the 0th to 24th hours. After the PTX treatment, a slight increase of 2% ± 1.1% in the intracellular Ca^2+^ level was observed (Figure 2E). In the MDA‐MB‐231 cell line, an increase of 11% ± 0.5% was observed after the 4‐AP treatment during the first 8 h; in the time interval from 8th to 24th hours, a decrease of approximately 9% ± 0.5% was observed. After the PTX treatment, a decrease of 13% ± 0.5% was detected. For combination treatment, an increase of 17% ± 2.3% was observed, a drastic decrease was not observed from the 8th to the 24th hours, and this increase was stable for 24 h (Figure 2F). The effect of the combination treatment on the intracellular Ca^+2^ concentration was significant, Tukey's results showed that the effects of every treatment group on intracellular Ca^+2^ concentration were significantly different from each other in both cell lines (for details, Data S2). The combination treatment resulted in a permanent increase in intracellular Ca^2+^ levels in both cell lines, with no decrease observed during the measurement period. This effect was distinct from that of 4‐AP. (Figure 2).

### 3.4 Transmembrane Potentials Were More Depolarized After Treatments

The changes in cell transmembrane potential of 4‐AP, PTX, and their combination used were determined with the transmembrane potential sensitive DiBAC_4_(3) fluorescent probe. When the membrane depolarizes, the dye is displaced inside the cell and binds to lipids or proteins, thereby increasing the fluorescence intensity. In the case of hyperpolarization, the fluorescence intensity decreases. Transmembrane potential measurements were taken at 6‐h intervals from the 15th minute after adding the drugs to determine the changes in the transmembrane potential after the separate and combined use of each drug, and the results are given in Figure 2C,D as a histogram.

For the MCF‐7 cell line, depolarization was observed for all treatment groups, 29% ± 3.4% for 4‐AP, 48% ± 5.4% for PTX, and 136% ± 4.8% for the combination treatment relative to control. 4‐AP treatment resulted in depolarization of the plasma membrane potential, which was proportional to changes in K^+^ concentration. While 4‐AP also increased intracellular Ca^2+^ levels, the changes in K^+^ and Ca^2+^ concentrations did not seem to exhibit an additive effect on membrane depolarization.

In MDA‐MB‐231, all treatments caused depolarization, an increase of the transmembrane potential was 60% ± 4.4% for 4‐AP, 114% ± 5.5% for PTX, and 231% ± 7.5% for the combination treatment. The treatments caused more depolarization in the MDA‐MB‐231 cell line than in the MCF‐7 cell line. For the MDA‐MB‐231 cell line, the depolarization observed after 4‐AP and the combination treatments seem to result from the increase in both K^+^ and Ca^2+^ concentrations.

For both cell lines, the source of the depolarization caused by PTX appears to be consistent with the increase in K^+^ concentration.

These results imply that for both cell lines, every treatment affected differently the transmembrane potential levels.

### 3.5 4‐AP and PTX Caused G_1_ Arrest in the MCF‐7 Cell Line While the Response of the MDA‐MB‐231 to PTX Was Different, the Combination Caused S Phase Arrest in Both Cell Lines

**FIGURE 3:**
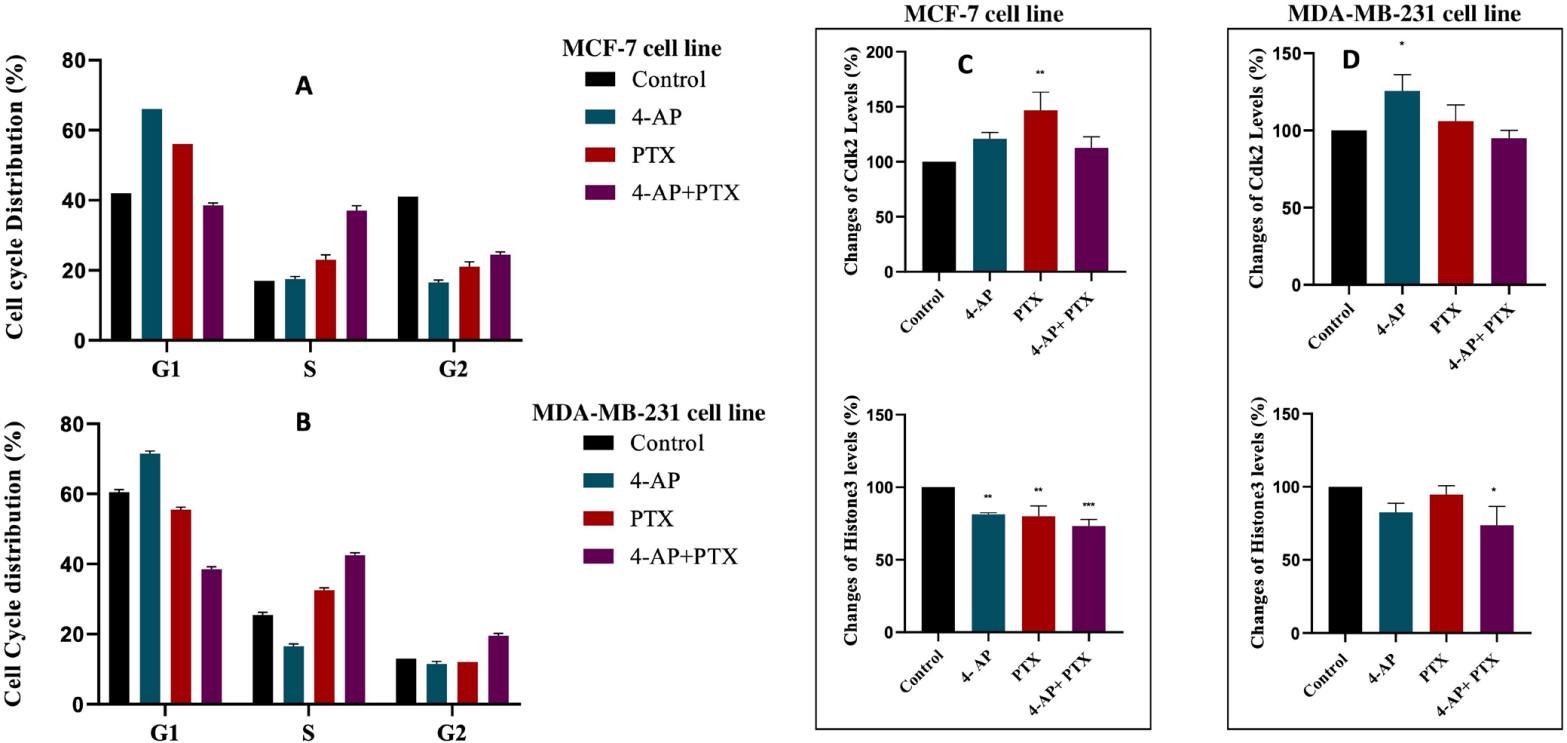
Determination of the cell cycle distribution. For detailed analyses, the cell cycle distribution was detected by a flow cytometer. In MCF‐7 cell line (A), the G1 arrest was observed after 4‐AP and PTX treatments. Additionally, PTX treatment caused a slight S‐phase arrest. The combination treatment caused the S phase arrest. In MDA‐MB‐231 cell line (B), 4‐AP caused G1 arrest, PTX caused S phase arrest, and the combination caused S phase and G2 arrest in MDA‐MB‐231 cell line. Determination of the cell cycle was also performed using a cell cycle assay kit by spectrophotometer. In MCF7 cell line (C), after 4‐AP, PTX, and the combination treatments, Cdk2 and Histone3 levels were determined by a cell cycle analysis kit. 4‐AP and PTX treatments caused an increase in Cdk2 levels. In Histone3 levels, there was no increase after all treatments [28]. In MDA‐MB 231 cell line (D), in Cdk2 level, 4‐AP caused an obvious increase, PTX caused a slight increase, and the combination treatment did not cause any change, in histone H3 levels. For all experiments, *n* ≥ 3, *p* < 0.05 was indicated *, *p* < 0.01 was indicated **, *p* < 0.001 was indicated *** (with respect to control).

Two cell cycle analyses were made. After incubation of each drug separately and in combination with cells for 24 h, changes in the cell cycle were recorded in flow cytometry using RNAse A and PI methods. Analysis of the results was performed using the Cell Quest program. Treatment of the 4‐AP caused the G_1_ arrest in the cell cycle for both cell lines, this result was expected. The G_1_ arrest was expected after the low‐dose treatment of the PTX. For the MCF‐7 cell line, PTX treatment caused G_1_ arrest and a slight cell accumulation in the S phase but for the MDA‐MB‐231 cell line after the PTX treatment, the S phase arrest was detected. Combination treatment caused the S phase arrest in MCF‐7 and a slight S phase arrest and G_2_/M arrest in the MDA‐MB‐231 cell line were observed (Figure 3A,B).

A second analysis was performed using a cell cycle assay kit which shows cell cycle arrest in histone H3 and cdk2 levels. The results of this kit were not as detailed as those of the flow cytometer. In the MCF‐7 cell line, after the 4‐AP, PTX, and the combination treatment the cdk2 levels showed an increase relative to control; 20.6% ± 6.1% for 4‐AP, 46.6% ± 16.5% for PTX, and 12.6% ± 10% for the combination treatment (Figure 3C) indicating a G_0_/G_1_ arrest for all treatment groups in MCF7 cell line. On the other hand, a decrease was observed in histone3 levels for all treatment groups; 18.6% ± 1.2% for 4‐AP, 20.0% ± 7.2% for PTX, and 26.6% ± 4.5% for combination treatment relative to control, implying that cell cycle did not progress to the G_2_/M phase in the MCF‐7 cell line (Figure 3C). For the MDA‐MB‐231 cell line, Cdk2 measurements showed an increase in 4‐AP (25.6% ± 10.5%) showing a G_0_/G1 arrest however no difference was observed for PTX treatment relative to control. A decrease of 5.0% ± 5.0% which was not statistically significant was observed for the combination treatment (Figure 3D). In histone H3 measurements, a decrease was observed for 4‐AP (17.3% ± 6.0%) and combination treatment (26.3% ± 13.1%) relative to control. These data must be considered with caution since a slight G2/M arrest was observed in flow cytometry measurements. Other methods to determine the cell cycle arrest are indicative.

From a statistical point of view, the results of the flow cytometer and the results of cdk2/H3 were compatible in the MCF‐7 cell line. In the MDA‐MB‐231 cell line, the results of the flow cytometer and the results of the cdk2/H3 were only compatible with the 4‐AP treatment group. For the measurements of Cdk2 and H3 levels, the results of one‐way ANOVA were significant, and the results of the multiple comparisons showed that for the changes in the Cdk2 level, PTX treatment was significantly different from the control group, indicating that it effectively increased Cdk2 level in the MCF‐7 cell line. In the changes in H3 levels, every treatment group was significantly different from the control group in the MCF‐7 cell line.

In the MDA‐MB‐231 cell line, from the results of multiple comparisons, the effects of 4‐AP treatment were significant relative to control and 4‐AP was more effective in the changes of Cdk2 level. In the changes of H3 levels, the results showed that 4‐AP and PTX were not significant and had no effect on the MDA‐MB‐231 cell line. The combination treatment showed a reduction in H3 levels in the MDA‐MB‐231 cell line.

## 4 Discussion

Paclitaxel has been used as an antineoplastic drug for a wide array of cancers. The major limitations of paclitaxel treatment are its side effects and acquired resistance to treatment [29]. Although there is ongoing research on novel, highly effective therapies for BC, overcoming resistance and thus maximizing the efficacy of paclitaxel is still a strong alternative.

This study aimed to use 4‐AP which is known to block voltage‐gated K^+^ channels thus halting the cell cycle at the G_1_ phase [27] in combination with paclitaxel to increase its effectiveness. Our previous study [28] with the MCF‐7 cell line gave promising results. PTX and 4‐AP acted synergistically on the MCF‐7 cell line blocking the cell cycle at the G_1_ phase as expected. Our goal was to replicate our previous results, determine if this increase in efficiency of PTX would be also valid for the metastatic cell line MDA‐MB‐231, and to determine the changes that would be evoked by 4‐AP namely changes in potassium and calcium concentrations and transmembrane potential that would give an insight for the estimation of the mechanism of this effect. A clonogenic assay was also carried out to determine whether this combination affected reproductive cell survival.

We first determined IC50 values for PTX and 4‐AP. A value of 7, 5 nM was found for PTX which is similar to that found by Jordan et al. for the HeLa cell line, 8 nM, [8]. An IC50 value was found for 4‐AP which is compatible with the value found by Chin et al. [13] for the U87 cell line. (See Data S1). Although different concentrations of 4‐AP and PTX were also tested to observe the efficiency of combination treatment and the impact of 4‐AP on PTX (Data not shown) the results were not significantly different than those used with IC50 values, so it proceeded with IC50 values. Combination treatment with IC50 values decreased survival to around 30% which we believe to be a promising result.

The colony formation ability of the surviving cells was determined by clonogenic assay. Separate treatments of 4‐ AP, PTX and the combination reduced colony number by 63.4% ± 2.24% (*p* < 0.0001), 65.5% ± 1.82% (*p* < 0.0001), and 72.7% ± 0.96% (*p* < 0.0001) respectively, relative to control in the MCF‐7 cell line; in the MDA‐MB‐231 cell line, the reduction of the colony number was 70.3% ± 1.58% (*p* < 0.0001) for 4‐AP, 76.0% ± 5.37% (*p* < 0.0001) for PTX and 85.9% ± 4.62% (*p* < 0.0001) for the combination treatment relative to control, and in the combination treatment this effect continued increasingly (Figure 1C,D). With these results, it can be said that the effects of the combination treatment on cell growth were not transient.

Apoptosis measurements for the combination treatment showed that the ratio of apoptotic cells was 44.8% ± 1.3% with respect to control in the case of the MCF‐7 cell line and 18.1% ± 0.4% in the MDA‐MB‐231 cell line. In the MCF‐7 cell line, combination treatment caused a significant increase in apoptotic cells which can be attributed to increased Ca^2+^ concentrations (Figure 2E). Considering that cell death was 70% in MCF‐7 and 60% in the MDA‐MB‐231 cell line it is reasonable to think that other cell death mechanisms may also be involved.

In both cell lines, 4‐AP treatment caused an increase in intracellular K^+^ ion concentrations as expected. PTX alone did not cause a noteworthy [K^+^] increase in the MCF‐7 cell line; however, it caused a significant increase in the MDA‐MB‐231 cell line. However, results for MDA‐MB‐231 must be considered with caution. First, it must be kept in mind that Kv channels are differentially expressed in MCF‐7 and MDA‐MB‐231 cell lines [30, 31] and second the Kd of fluorescent probe PBFI/AM decreases under high Na^+^ concentrations causing higher K^+^ measurements. Voltage‐gated Na^+^ channel (VGSC) is shown to be highly expressed in metastatic cancerous cells [31, 32] and PTX was seen to increase VGSC (Na_V_1.7) in dorsal root ganglion cells [33] that might have also been the case for MDA‐MB‐231. Also, PTX is seen to block potassium channels in neuronal and cardiac cells (Wu et al., 2022; Kitamura et al., 2015) which may be relevant for BC cell lines.

It is obvious that changes in K^+^ ion concentration would affect the transmembrane potential. 4‐AP, PTX, and the combination treatment resulted in depolarization (Figure 2A,B.) in both cell lines, in accordance with the increase in [K^+^]_i_ as stated previously. Application of both 4‐AP and PTX caused a sustained depolarization which may explain in part the synergistic effect of this combination namely the blockage of the cell cycle (Figure 3).

The changes in the cell cycle dynamics were also evaluated. With the effect of 4‐AP in MCF‐7 and MDA‐MB‐231 cell lines, G_1_ phase arrest was observed. This result was expected because 4‐AP leads to cell membrane depolarization and since hyperpolarization is needed for the S phase, depolarization prevents the progression from the G_1_ phase to the S phase during the cell cycle [26, 34]. PTX treatment caused a G_1_ phase arrest in the MCF7 cell line. In this study, a low dose of PTX was chosen and previous studies showed that a low dose value of PTX caused the G_1_ phase arrest (in accordance with the results of Pushkarev et al. [35]). Giannakakou et al. showed that a 3 nM concentration of PTX caused complete inhibition of cell growth and this concentration value led to G_1_ arrest in the A549 cell line [7]. In the MDA‐MB‐231 cell line, PTX did not cause the G_1_ phase arrest, it caused only slight cell accumulation in the S phase according to flow cytometric measurements. After the combination treatment, in the MCF‐7 cell line, S‐phase arrest was observed. In the MDA‐MB‐231 cell line, the S phase arrest and a slight cell accumulation in the G_2_/M phase were observed with flow cytometry. A reason for cell accumulation in the S phase can be the halted DNA replication—detailed analysis such as BrdU labeling is needed for the determination of the DNA content of the cells and cell cycle analysis.

Ca^2+^ signaling is altered in cancer. A higher expression of calcium channels, pumps, GPCRs, and TRP channels is observed which seems to be necessary for cancer progression [36]. On the other hand, it is known that a sustained Ca^2+^ would trigger cell death. Our data showed that Ca^2+^ was increased upon 4‐AP treatment at IC_50_ values in the MCF‐7 cell line which is in accordance with the depolarization due to the blockage of voltage‐gated K^+^ channels (VGPC) and the consequent activation of voltage‐gated Ca^2+^ channels as stated previously by Kim et al. in the HEPG2 cell line [23]. However, no increase in calcium was observed in MDA‐MB‐231 cells upon 4‐AP treatment at the end of the 24th hour. Pera et al. stated that voltage‐gated calcium channel expression (Ca_v_3.2) was high for the MCF‐7 cell line while it was undetectable in the MDA‐MB‐231 cell line [37]. Also, Payne et al. observed no change in Ca^2+^ when the K^+^ channel was blocked in a triple‐negative cell line [38]. These data seem to be in accordance with our results. Interestingly combination treatment on the other hand resulted in an elevated Ca^2+^ concentration which correlates with a higher apoptotic ratio and needs further investigation.

This study aimed to determine whether 4‐AP could potentiate the effect of paclitaxel. We have shown that 4‐AP does indeed increase the effectiveness of PTX in 24 h and as the colony formation showed, in the long term. We have conducted studies on K^+^, Ca^2+^ concentrations, cell cycle, and membrane potential to give a standpoint for the probable mechanism of this combination. We acknowledge that further studies are required for each parameter. Drug repurposing is an important and efficient alternative for novel drug discovery and blocking ion channels especially potassium and calcium is a promising strategy [39] for the development of new combination treatments. We believe that the combination of 4‐AP and PTX would be a promising candidate and deserves further studies both in in vivo and in vitro.

## Author Contributions

Conceptualization: Esra M. Cüce‐Aydoğmuş, G. Ayşe İnhan‐Garip. Data curation: Esra M. Cüce‐Aydoğmuş. Investigation: Esra M. Cüce‐Aydoğmuş. Methodology: Esra M. Cüce‐Aydoğmuş. Project administration: Esra M. Cüce‐Aydoğmuş, G. Ayşe İnhan‐Garip. Writing – original draft: Esra M. Cüce‐Aydoğmuş. Writing – review and editing: G. Ayşe İnhan‐Garip. Supervision: G. Ayşe İnhan‐Garip.

## Ethics Statement

The authors have nothing to report.

## Conflicts of Interest

The authors declare no conflicts of interest.

## Supporting information


Data S1.

Data S2.

## Data Availability

The data that support the findings of this study are available from the corresponding author upon reasonable request.
