# Supplementary material for: Investigation of the Effects of Blocking Potassium Channels With 4‐Aminopyridine on Paclitaxel Activity in Breast Cancer Cell Lines
Source: Cancer Rep (Hoboken). 2024 Dec 8;7(12):e70072. doi: 10.1002/cnr2.70072 (PMC11625685; doi:10.1002/cnr2.70072)
Supplement: Supplementary file 1 — Data S1. [file CNR2-7-e70072-s001.docx]

**Supplementary 1**

To determine the IC_50_ values of PTX and 4-AP, the cells were counted by trypan blue method in a hemocytometer after treatments for 24h. For MCF-7 cell line, IC_50_ value of PTX was determined 7.5 nM (A), and for MDA-MB-231, IC_50_ value was determined 8 nM (C). For combination treatments, 7.5 nM was chosen for PTX dose. For 4-AP, IC_50_ value was determined at 4 mM for both cell lines (MCF-7 in B, MDA-MB-231 in D). For statistical evaluation, one- way ANOVA was performed and as post hoc analysis, Dunnet’s test were performed.


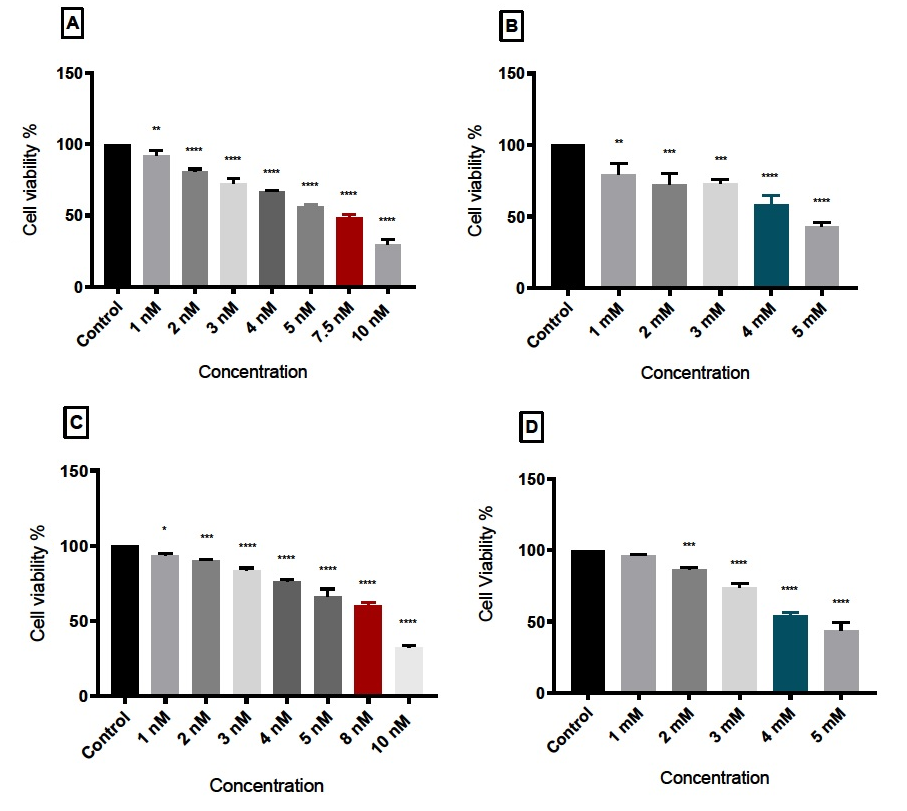


**The details of Dunnet’s test analyses were given in the table.**

| **MCF-7, IC_50_ value of PTX** |  |  |  |  |  |
| --- | --- | --- | --- | --- | --- |
| **ANOVA summary** |  |  |  |  |  |
| F | 287,9 | |  |  |  |
| P value | <0,0001 | |  |  |  |
| **P value summary** | ******** | |  |  |  |
| Significant diff. among means (P < 0.05)? | Yes | |  |  |  |
| R square | 0,9921 | |  |  |  |
| **Alpha** | **0,05** |  |  |  |  |
| **Dunnett's multiple comparisons test** | **Mean Diff,** | **95,00% CI of diff,** | **Significant?** | **Summary** | **Adjusted P Value** |
| Control vs. 1 nM | 7,667 | 2,004 to 13,33 | Yes | ** | 0,0063 |
| Control vs. 2 nM | 19,00 | 13,34 to 24,66 | Yes | **** | <0,0001 |
| Control vs. 3 nM | 27,33 | 21,67 to 33,00 | Yes | **** | <0,0001 |
| Control vs. 4 nM | 33,33 | 27,67 to 39,00 | Yes | **** | <0,0001 |
| Control vs. 5 nM | 43,33 | 37,67 to 49,00 | Yes | **** | <0,0001 |
| Control vs. 7.5 nM | 51,33 | 45,67 to 57,00 | Yes | **** | <0,0001 |
| Control vs. 10 nM | 70,33 | 64,67 to 76,00 | Yes | **** | <0,0001 |

| **MCF-7, IC_50_ value of 4-AP** |  |  |  |  |  |
| --- | --- | --- | --- | --- | --- |
| **ANOVA summary** |  |  |  |  |  |
| F | 39,83 | |  |  |  |
| P value | <0,0001 | |  |  |  |
| **P value summary** | ******** | |  |  |  |
| Significant diff. among means (P < 0.05)? | Yes | |  |  |  |
| R square | 0,9432 | |  |  |  |
| **Alpha** | **0,05** |  |  |  |  |
| **Dunnett's multiple comparisons test** | **Mean Diff,** | **95,00% CI of diff,** | **Significant?** | **Summary** | **Adjusted P Value** |
| Control vs. 1 mM | 20,33 | 7,861 to 32,81 | Yes | ** | 0,0021 |
| Control vs. 2 mM | 27,33 | 14,86 to 39,81 | Yes | *** | 0,0002 |
| Control vs. 3 mM | 27,00 | 14,53 to 39,47 | Yes | *** | 0,0002 |
| Control vs. 4 mM | 41,33 | 28,86 to 53,81 | Yes | **** | <0,0001 |
| Control vs. 5 mM | 56,67 | 44,19 to 69,14 | Yes | **** | <0,0001 |

| **MDA-MB-231, IC_50_ value of** **PTX** | |  |  |  |  |
| --- | --- | --- | --- | --- | --- |
| **ANOVA summary** | |  |  |  |  |
| F | | 281,1 |  |  |  |
| P value | | <0,0001 |  |  |  |
| **P value summary** | | ******** |  |  |  |
| Significant diff. among means (P < 0.05)? | | Yes |  |  |  |
| R square | | 0,9919 |  |  |  |
| **Alpha** | **0,05** |  |  |  |  |
| **Dunnett's multiple comparisons test** | **Mean Diff,** | **95,00% CI of diff,** | **Significant?** | **Summary** | **Adjusted P Value** |
| Control vs. 1 nM | 6,667 | 1,240 to 12,09 | Yes | * | 0,0133 |
| Control vs. 2 nM | 10,00 | 4,573 to 15,43 | Yes | *** | 0,0004 |
| Control vs. 3 nM | 16,33 | 10,91 to 21,76 | Yes | **** | <0,0001 |
| Control vs. 4 nM | 23,67 | 18,24 to 29,09 | Yes | **** | <0,0001 |
| Control vs. 5 nM | 34,00 | 28,57 to 39,43 | Yes | **** | <0,0001 |
| Control vs. 8 nM | 40,00 | 34,57 to 45,43 | Yes | **** | <0,0001 |
| Control vs. 10 nM | 67,67 | 62,24 to 73,09 | Yes | **** | <0,0001 |

| **MDA-MB-231, IC_50_ value of 4-AP** |  |  |  |  |  |
| --- | --- | --- | --- | --- | --- |
| **ANOVA summary** |  |  |  |  |  |
| F | 205,3 | |  |  |  |
| P value | <0,0001 | |  |  |  |
| **P value summary** | ******** | |  |  |  |
| Significant diff. among means (P < 0.05)? | Yes | |  |  |  |
| R square | 0,9884 | |  |  |  |
| **Alpha** | **0,05** |  |  |  |  |
| **Dunnett's multiple comparisons test** | **Mean Diff,** | **95,00% CI of diff,** | **Significant?** | **Summary** | **Adjusted P Value** |
| Control vs. 1 mM | 3,333 | -3,226 to 9,893 | No | ns | 0,4735 |
| Control vs. 2 mM | 13,67 | 7,107 to 20,23 | Yes | *** | 0,0003 |
| Control vs. 3 mM | 26,33 | 19,77 to 32,89 | Yes | **** | <0,0001 |
| Control vs. 4 mM | 46,00 | 39,44 to 52,56 | Yes | **** | <0,0001 |
| Control vs. 5 mM | 56,00 | 49,44 to 62,56 | Yes | **** | <0,0001 |
