# Supplementary material for: Investigation of the Effects of Blocking Potassium Channels With 4‐Aminopyridine on Paclitaxel Activity in Breast Cancer Cell Lines
Source: Cancer Rep (Hoboken). 2024 Dec 8;7(12):e70072. doi: 10.1002/cnr2.70072 (PMC11625685; doi:10.1002/cnr2.70072)
Supplement: Supplementary file 2 — Data S2. [file CNR2-7-e70072-s002.docx]

**Supplementary 2:** **For multiple comparisons, Tukey’s test was performed when the variable number was more than 2. Tukey's results were given in sequence in manuscript.**

| **Colony forming assay for MCF-7**  ANOVA summary |  |  |
| --- | --- | --- |
| F |  | 7541 |
| P value |  | <0,0001 |
| P value summary |  | **** |
| Significant diff. among means (P < 0.05)? |  | Yes |
| R square |  | 0,9996 |

| Alpha | 0,05 |  |  |  |  |
| --- | --- | --- | --- | --- | --- |
|  |  |  |  |  |  |
| Tukey's multiple comparisons test | Mean Diff, | 95,00% CI of diff, | Significant? | Summary | Adjusted P Value |
| Control vs. 4-AP | 63,00 | 61,19 to 64,81 | Yes | **** | <0,0001 |
| Control vs. PTX | 65,33 | 63,52 to 67,15 | Yes | **** | <0,0001 |
| Control vs. 4-AP+ PTX | 77,00 | 75,19 to 78,81 | Yes | **** | <0,0001 |
| 4-AP vs. PTX | 2,333 | 0,5202 to 4,146 | Yes | * | 0,0142 |
| 4-AP vs. 4-AP+ PTX | 14,00 | 12,19 to 15,81 | Yes | **** | <0,0001 |
| PTX vs. 4-AP+ PTX | 11,67 | 9,854 to 13,48 | Yes | **** | <0,0001 |

**Colony Forming assay for MDA-MB-231**

| ANOVA summary |  |
| --- | --- |
| F | 167,3 |
| P value | <0,0001 |
| P value summary | **** |
| Significant diff. among means (P < 0.05)? | Yes |
| R square | 0,9843 |

| Alpha | 0,05 |  |  |  |  |
| --- | --- | --- | --- | --- | --- |
|  |  |  |  |  |  |
| Tukey's multiple comparisons test | Mean Diff, | 95,00% CI of diff, | Significant? | Summary | Adjusted P Value |
| Control vs. 4-AP | 69,67 | 66,21 to 73,13 | Yes | **** | <0,0001 |
| Control vs. PTX | 73,67 | 70,21 to 77,13 | Yes | **** | <0,0001 |
| Control vs. 4-AP+ PTX | 83,67 | 80,21 to 87,13 | Yes | **** | <0,0001 |
| 4-AP vs. PTX | 4,000 | 0,5411 to 7,459 | Yes | * | 0,0249 |
| 4-AP vs. 4-AP+ PTX | 14,00 | 10,54 to 17,46 | Yes | **** | <0,0001 |
| PTX vs. 4-AP+ PTX | 10,00 | 6,541 to 13,46 | Yes | **** | <0,0001 |

**For the changes in intracellular K^+^ concentration in MCF-7 cell line.**

| ANOVA summary |  |
| --- | --- |
| F | 18,88 |
| P value | 0,0005 |
| P value summary | *** |
| Significant diff. among means (P < 0.05)? | Yes |
| R square | 0,8762 |

| Alpha | 0,05 |  |  |  |  |
| --- | --- | --- | --- | --- | --- |
|  |  |  |  |  |  |
| Tukey's multiple comparisons test | Mean Diff, | 95,00% CI of diff, | Significant? | Summary | Adjusted P Value |
| Control vs. 4-AP | -30,33 | -48,59 to -12,08 | Yes | ** | 0,0031 |
| Control vs. PTX | -21,67 | -39,92 to -3,410 | Yes | * | 0,0218 |
| Control vs. 4-AP+ PTX | -41,33 | -59,59 to -23,08 | Yes | *** | 0,0004 |
| 4-AP vs. PTX | 8,667 | -9,590 to 26,92 | No | ns | 0,4697 |
| 4-AP vs. 4-AP+ PTX | -11,00 | -29,26 to 7,256 | No | ns | 0,2890 |
| PTX vs. 4-AP+ PTX | -19,67 | -37,92 to -1,410 | Yes | * | 0,0354 |

**For the changes in intracellular K^+^ concentration in MDA- MB-231 cell line.**

| ANOVA summary |  |
| --- | --- |
| F | 167,3 |
| P value | <0,0001 |
| P value summary | **** |
| Significant diff. among means (P < 0.05)? | Yes |
| R square | 0,9843 |

| Alpha | 0,05 |  |  |  |  |
| --- | --- | --- | --- | --- | --- |
|  |  |  |  |  |  |
| Tukey's multiple comparisons test | Mean Diff, | 95,00% CI of diff, | Significant? | Summary | Adjusted P Value |
| Control vs. 4-AP | -28,67 | -69,25 to 11,92 | No | ns | 0,1865 |
| Control vs. PTX | -258,7 | -299,3 to -218,1 | Yes | **** | <0,0001 |
| Control vs. 4-AP+ PTX | -85,00 | -125,6 to -44,42 | Yes | *** | 0,0007 |
| 4-AP vs. PTX | -230,0 | -270,6 to -189,4 | Yes | **** | <0,0001 |
| 4-AP vs. 4-AP+ PTX | -56,33 | -96,92 to -15,75 | Yes | ** | 0,0093 |
| PTX vs. 4-AP+ PTX | 173,7 | 133,1 to 214,3 | Yes | **** | <0,0001 |

**For the changes in intracellular Ca levels, the results at 24th hours were taken into account for statistical analyses.**

**For MCF-7 cell line:**

| ANOVA summary |  |
| --- | --- |
| F | 224,7 |
| P value | <0,0001 |
| P value summary | **** |
| Significant diff. among means (P < 0.05)? | Yes |
| R square | 0,9883 |

| Alpha | 0,05 |  |  |  |  |
| --- | --- | --- | --- | --- | --- |
|  |  |  |  |  |  |
| Tukey's multiple comparisons test | Mean Diff, | 95,00% CI of diff, | Significant? | Summary | Adjusted P Value |
| Control vs. 4-AP | 0,08667 | -0,03782 to 0,2112 | No | ns | 0,1949 |
| Control vs. PTX | -0,5633 | -0,6878 to -0,4388 | Yes | **** | <0,0001 |
| Control vs. 4-AP+ PTX | 0,4300 | 0,3055 to 0,5545 | Yes | **** | <0,0001 |
| 4-AP vs. PTX | -0,6500 | -0,7745 to -0,5255 | Yes | **** | <0,0001 |
| 4-AP vs. 4-AP+ PTX | 0,3433 | 0,2188 to 0,4678 | Yes | **** | <0,0001 |
| PTX vs. 4-AP+ PTX | 0,9933 | 0,8688 to 1,118 | Yes | **** | <0,0001 |

**For MDA-MB-231 cell line:**

| ANOVA summary |  |
| --- | --- |
| F | 131,9 |
| P value | <0,0001 |
| P value summary | **** |
| Significant diff. among means (P < 0.05)? | Yes |
| R square | 0,9802 |

| Alpha | 0,05 |  |  |  |  |
| --- | --- | --- | --- | --- | --- |
|  |  |  |  |  |  |
| Tukey's multiple comparisons test | Mean Diff, | 95,00% CI of diff, | Significant? | Summary | Adjusted P Value |
| Control vs. 4-AP | -0,5767 | -0,7138 to -0,4396 | Yes | **** | <0,0001 |
| Control vs. PTX | -0,6867 | -0,8238 to -0,5496 | Yes | **** | <0,0001 |
| Control vs. 4-AP+ PTX | -0,07333 | -0,2104 to 0,06378 | No | ns | 0,3773 |
| 4-AP vs. PTX | -0,1100 | -0,2471 to 0,02712 | No | ns | 0,1221 |
| 4-AP vs. 4-AP+ PTX | 0,5033 | 0,3662 to 0,6404 | Yes | **** | <0,0001 |
| PTX vs. 4-AP+ PTX | 0,6133 | 0,4762 to 0,7504 | Yes | **** | <0,0001 |

**The changes in transmembrane potential in MCF-7 cell line:**

| ANOVA summary |  |
| --- | --- |
| F | 646,1 |
| P value | <0,0001 |
| P value summary | **** |
| Significant diff. among means (P < 0.05)? | Yes |
| R square | 0,9959 |

| Alpha | 0,05 |  |  |  |  |
| --- | --- | --- | --- | --- | --- |
|  |  |  |  |  |  |
| Tukey's multiple comparisons test | Mean Diff, | 95,00% CI of diff, | Significant? | Summary | Adjusted P Value |
| Control vs. 4-AP | -29,00 | -39,44 to -18,56 | Yes | **** | <0,0001 |
| Control vs. PTX | -48,00 | -58,44 to -37,56 | Yes | **** | <0,0001 |
| Control vs. 4-AP+ PTX | -136,0 | -146,4 to -125,6 | Yes | **** | <0,0001 |
| 4-AP vs. PTX | -19,00 | -29,44 to -8,561 | Yes | ** | 0,0018 |
| 4-AP vs. 4-AP+ PTX | -107,0 | -117,4 to -96,56 | Yes | **** | <0,0001 |
| PTX vs. 4-AP+ PTX | -88,00 | -98,44 to -77,56 | Yes | **** | <0,0001 |

**The changes in transmembrane potential in MDA-MB-231 cell line:**

| ANOVA summary |  |
| --- | --- |
| F | 926,3 |
| P value | <0,0001 |
| P value summary | **** |
| Significant diff. among means (P < 0.05)? | Yes |
| R square | 0,9971 |

| Alpha | 0,05 |  |  |  |  |
| --- | --- | --- | --- | --- | --- |
|  |  |  |  |  |  |
| Tukey's multiple comparisons test | Mean Diff, | 95,00% CI of diff, | Significant? | Summary | Adjusted P Value |
| Control vs. 4-AP | -60,00 | -73,46 to -46,54 | Yes | **** | <0,0001 |
| Control vs. PTX | -114,3 | -127,8 to -100,9 | Yes | **** | <0,0001 |
| Control vs. 4-AP+ PTX | -213,0 | -226,5 to -199,5 | Yes | **** | <0,0001 |
| 4-AP vs. PTX | -54,33 | -67,79 to -40,88 | Yes | **** | <0,0001 |
| 4-AP vs. 4-AP+ PTX | -153,0 | -166,5 to -139,5 | Yes | **** | <0,0001 |
| PTX vs. 4-AP+ PTX | -98,67 | -112,1 to -85,21 | Yes | **** | <0,0001 |

**The changes in CDK2 levels in MCF-7 cell line:**

| ANOVA summary |  |
| --- | --- |
| F | 11,37 |
| P value | 0,0030 |
| P value summary | ** |
| Significant diff. among means (P < 0.05)? | Yes |
| R square | 0,8100 |

| Alpha | 0,05 |  |  |  |  |
| --- | --- | --- | --- | --- | --- |
|  |  |  |  |  |  |
| Tukey's multiple comparisons test | Mean Diff, | 95,00% CI of diff, | Significant? | Summary | Adjusted P Value |
| Control vs. 4-AP | -20,67 | -47,14 to 5,805 | No | ns | 0,1344 |
| Control vs. PTX | -46,67 | -73,14 to -20,19 | Yes | ** | 0,0022 |
| Control vs. 4-AP+ PTX | -12,67 | -39,14 to 13,81 | No | ns | 0,4635 |
| 4-AP vs. PTX | -26,00 | -52,47 to 0,4719 | No | ns | 0,0542 |
| 4-AP vs. 4-AP+ PTX | 8,000 | -18,47 to 34,47 | No | ns | 0,7708 |
| PTX vs. 4-AP+ PTX | 34,00 | 7,528 to 60,47 | Yes | * | 0,0143 |

**The changes in Histone3 levels in MCF-7 cell line:**

| ANOVA summary |  |
| --- | --- |
| F | 21,31 |
| P value | 0,0004 |
| P value summary | *** |
| Significant diff. among means (P < 0.05)? | Yes |
| R square | 0,8888 |

| Alpha | 0,05 |  |  |  |  |
| --- | --- | --- | --- | --- | --- |
|  |  |  |  |  |  |
| Tukey's multiple comparisons test | Mean Diff, | 95,00% CI of diff, | Significant? | Summary | Adjusted P Value |
| Control vs. 4- AP | 18,67 | 7,446 to 29,89 | Yes | ** | 0,0031 |
| Control vs. PTX | 20,00 | 8,779 to 31,22 | Yes | ** | 0,0020 |
| Control vs. 4-AP+ PTX | 26,67 | 15,45 to 37,89 | Yes | *** | 0,0003 |
| 4- AP vs. PTX | 1,333 | -9,888 to 12,55 | No | ns | 0,9799 |
| 4- AP vs. 4-AP+ PTX | 8,000 | -3,221 to 19,22 | No | ns | 0,1812 |
| PTX vs. 4-AP+ PTX | 6,667 | -4,554 to 17,89 | No | ns | 0,2990 |

**The changes in CDK2 levels in MDA-MB-231 cell line:**

| ANOVA summary |  |
| --- | --- |
| F | 8,765 |
| P value | 0,0066 |
| P value summary | ** |
| Significant diff. among means (P < 0.05)? | Yes |
| R square | 0,7667 |

| Alpha | 0,05 |  |  |  |  |
| --- | --- | --- | --- | --- | --- |
|  |  |  |  |  |  |
| Tukey's multiple comparisons test | Mean Diff, | 95,00% CI of diff, | Significant? | Summary | Adjusted P Value |
| Control vs. 4-AP | -25,67 | -46,23 to -5,106 | Yes | * | 0,0167 |
| Control vs. PTX | -6,000 | -26,56 to 14,56 | No | ns | 0,7880 |
| Control vs. 4-AP+ PTX | 5,000 | -15,56 to 25,56 | No | ns | 0,8620 |
| 4-AP vs. PTX | 19,67 | -0,8939 to 40,23 | No | ns | 0,0608 |
| 4-AP vs. 4-AP+ PTX | 30,67 | 10,11 to 51,23 | Yes | ** | 0,0061 |
| PTX vs. 4-AP+ PTX | 11,00 | -9,561 to 31,56 | No | ns | 0,3771 |

**The changes in Histone3 levels in MDA-MB-231 cell line:**

| ANOVA summary |  |
| --- | --- |
| F | 6,919 |
| P value | 0,0130 |
| P value summary | * |
| Significant diff. among means (P < 0.05)? | Yes |
| R square | 0,7218 |

| Alpha | 0,05 |  |  |  |  |
| --- | --- | --- | --- | --- | --- |
|  |  |  |  |  |  |
| Tukey's multiple comparisons test | Mean Diff, | 95,00% CI of diff, | Significant? | Summary | Adjusted P Value |
| Control vs. 4-AP | 17,33 | -3,088 to 37,75 | No | ns | 0,0990 |
| Control vs. PTX | 5,333 | -15,09 to 25,75 | No | ns | 0,8361 |
| Control vs. 4-AP+ PTX | 26,33 | 5,912 to 46,75 | Yes | * | 0,0140 |
| 4-AP vs. PTX | -12,00 | -32,42 to 8,422 | No | ns | 0,3070 |
| 4-AP vs. 4-AP+ PTX | 9,000 | -11,42 to 29,42 | No | ns | 0,5268 |
| PTX vs. 4-AP+ PTX | 21,00 | 0,5785 to 41,42 | Yes | * | 0,0440 |
